# Atrial fibrillation in the elderly population: Challenges and management considerations

**DOI:** 10.1002/joa3.12580

**Published:** 2021-06-24

**Authors:** Mohammed Salih, Osama Abdel‐Hafez, Ramzi Ibrahim, Rajiv Nair

**Affiliations:** ^1^ Department of Internal Medicine Cardiovascular Disease St Joseph Mercy Oakland Hospital Pontiac MI USA; ^2^ Departmen of Internal Medicine University of Arizona Tucson AZ USA

**Keywords:** anticoagulation, atrial fibrillation, rate control, rhythm control, stroke

## Abstract

**Importance:**

Atrial fibrillation is the most clinically significant arrhythmia in humans when viewed both from a global and also a national perspective. In the United States, approximately 2.7‐6.1 million people are estimated to have atrial fibrillation. With the aging of the population, this prevalence is on an increasing trend and remains an obstacle to cardiovascular health despite significant advancements specific to cardiovascular disease management.

**Observation:**

In this specific group of patients, healthcare utilization is a concern from the public health perspective. Unfortunately, misconceptions dominate clinical decision making; for instance, the avoidance of safe and effective anticoagulation strategies in patients at the highest risk for embolic strokes continues to be widespread in clinical practice and is often based on a skewed assessment of risk versus benefit. Also, when there are contraindications to standard interventions for atrial fibrillation, a clear and nuanced understanding of second‐ and third‐line interventions with proven benefit is often lacking.

**Conclusions and Relevance:**

An individualized approach should be followed by physicians when managing atrial fibrillation in the elderly patient, taking into consideration the risk of complications, particularly the embolic stroke and the availability of treatment options for stroke prevention whether through pharmacological anticoagulation or left atrial appendage occluding devices. The following review sets out to clarify these issues.

## Graphical abstract

Atrial arrhythmia is the most common arrhythmia in the elderly population, and its incidence is increasing. This is particularly important as under treatment and avoidance of anticoagulation can lead to serious consequences and complications.

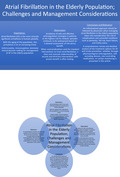

## 1 INTRODUCTION

Atrial fibrillation (AF) is usually, but not exclusively, associated with underlying cardiac disease. While rheumatic heart disease continues to be an important cause of AF in the developing world, it is becoming increasingly rare in Western societies. This review will primarily focus on nonrheumatic AF.

AF is a public health problem that continues to expand. The rising prevalence and incidence worldwide impose a health burden that requires an aggressive focus on mitigating risk factors, in addition to improving disease management. AF is common in the elderly; in this population, in whom comorbidities are widespread, a tailored approach to adequate management is required in order to provide maximum benefit, improve quality‐of‐life, and minimize risk. Hemodynamic changes resulting in atrial hypertension, morphologic changes showing fibrosis of the atria, and neurohumoral changes have all been described in nonrheumatic AF.

### 1.1 Epidemiology and risk factors

A systematic review conducted in 2010 on the number of individuals with AF worldwide reported a prevalence of 33.5 million, while in the United States, there were approximately a prevalence 5.2 million in 2010 with a projected increase to a prevalence of 12.1 million in 2030.^1^, ^2^ This prevalence is steadily rising as the population ages.^3^, ^4^ These numbers do not take into account subclinical AF, detected incidentally using implantable cardiac devices such as pacemakers, implantable cardioverter defibrillators (ICDs), and loop recorders. For example, in the ASSERT study, which included 2580 patients with pacemakers or ICDs above the age of 65 years and had no history of AF, 10% of patients had subclinical AF at 3 months and 35% at 2.5 years.^5^ The true prevalence of AF in the United States is therefore unknown.

The prevalence is greatly dependent on factors such as age, sex, ethnicity, and obesity. AF appears to be more common in men. It appears to be more prevalent in the Caucasian population than in African Americans.^3^

**FIGURE 1:**
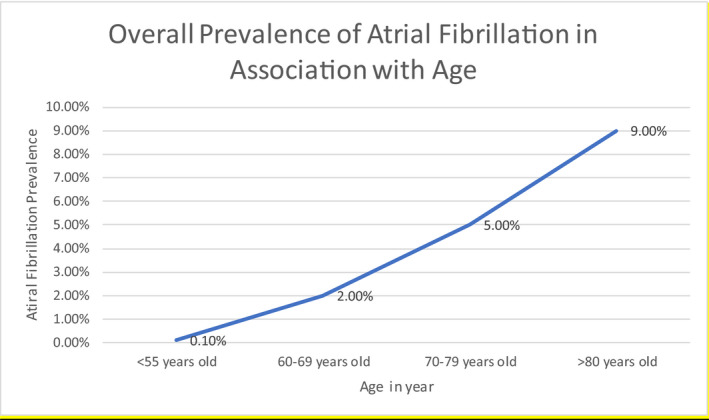
Prevalence of atrial fibrillation in association with age

AF is quite unusual in infants and young children in the absence of structural/congenital heart disease. In the developed world, age has much to do with the occurrence of AF. As seen In the ATRIA study, out of 1.89 million subjects, the prevalence of AF for individuals under 55 years was 0.1%.^4^ The risk of AF increases exponentially with aging; in the Framingham Heart Study, participants who were at least 40 years of age and had no history of AF had a 26% and 23% chance of future AF for men and women, respectively. Figure 1 shows estimated prevalence in accordance with age.

There are associated risk factors that significantly increase the risk of developing AF^6^, ^7^ (Table 1). In general, the risk of developing AF increases with any heart disease.^8^, ^9^ Among the most common diseases are hypertension, coronary artery disease, and heart failure (HF).^10^, ^11^ Valvular disease such as stenosis or regurgitation is another contributing factor to the development of AF by increasing atrial pressure and/or stretch.^12^ Twenty percent of adults who have had long‐standing atrial septal defects will develop AF.^13^ Pathologies that increase the right ventricular afterload, such as pulmonary emboli, chronic obstructive pulmonary disease, and obstructive sleep apnea, can ultimately increase the risk of AF.^14^, ^15^, ^16^, ^17^, ^18^, ^19^ According to the Framingham Heart Study, per one unit of BMI increase, the risk of AF increases by 5%.^20^, ^21^ A few other known risk factors for the development of AF are thyroid disease, metabolic syndrome, chronic kidney disease,^22^ and the stress of surgery or infection.^23^, ^24^, ^25^ Inflammation within the atria due to systemic mediators or localized processes can contribute to further worsening of conduction within the atrial tissue, leading to a higher susceptibility of AF.^25^

**TABLE 1:** Risk factors for atrial fibrillation with their corresponding hazard ratio

Risk factor	The hazard ratio for developing AF
Age	
45–54 y old	2.40
55–64 y old	4.65
65–74 y old	8.19
≥75	16.37
Male	1.32
Heart failure	1.72
Hypertension	1.31
Diabetes	1.11
Coronary artery disease	1.21
Chronic kidney disease	1.23
Obstructive sleep apnea	1.21
Current smoking	1.08
Moderate‐heavy drinking	1.05
Left ventricular hypertrophy on echocardiogram	1.36

### 1.2 Classification of AF

AF can be classified under different subtypes as defined by the 2014 American Heart Association guidelines^26^, ^27^ (Table 2). Paroxysmal AF is defined as AF that terminates spontaneously or by intervention (cardioversion) within 7 days. Most patients who have subclinical AF also are categorized under paroxysmal AF. Subclinical AF is typically diagnosed via ambulatory cardiac monitoring since the episodes of AF can occur randomly and often lack symptomology. Persistent AF is a failure of the arrhythmia to terminate within a 7‐day period. Long‐standing persistent AF is when it lasts for over 12 months, and permanent AF is a term used when rate control is the preferred long‐term strategy for managing the AF following a discussion between the clinician and the patient.^26^, ^27^

**TABLE 2:** Classification of atrial fibrillation

Classification	Definition
Paroxysmal AF	Arrhythmia terminates spontaneously or with intervention within 7 d
Persistent AF	Failure to terminate arrhythmia within 7 d
Long‐standing persistent AF	Persistent AF over 12 mo
Permanent AF	Joint decision between physician and patient to no longer restore or maintain sinus rhythm

### 1.3 Clinical presentation and diagnosis

AF may be asymptomatic or symptomatic. When asymptomatic, the initial presentation may be in the setting of an embolic event or during a medical evaluation for a different reason. Embolic stroke is one of the most catastrophic sequelae of AF. In fact, >15% of all patients who have experienced a stroke have had a history of AF.^28^ Crystal–AF trial showed that using extended implantable cardiac monitoring can detect subclinical AF in up to 30% at 36 months, in patients with cryptogenic stroke. The trial included 441 patients who were older than 40 years and diagnosed with cryptogenic stroke without identifying AF during a complete diagnostic evaluation up to 24 hours after their stroke presentation.^29^ Additionally, another study by Ziegler et al was conducted that followed patients who had an intracardiac monitor placed for the purpose of AF detection following cryptogenic stroke.^30^ They were monitored for up to 2 years to detect the frequency of AF episodes. In conclusion, this study showed that one in every five patients was detected to have at least one episode of AF (>2 minutes long). Conventional external ambulatory monitoring would not have been able to detect this same amount of AF episodes when compared to the insertion of an implantable cardiac monitor.

When symptomatic, the common symptoms are irregular palpitations, chest tightness, fatigue, and shortness of breath. As these symptoms, particularly fatigue, are very nonspecific, the diagnosis can be very difficult, particularly in the setting of infrequent paroxysms of AF.

Ultimately, the diagnosis is made on the basis of the electrocardiogram, which typically shows the absence of distinct organized atrial activity, the presence of fibrillatory waves, and the irregularly irregular nature of the cardiac rhythm. A standard 12‐lead ECG taken during an episode of AF is diagnostic. Extended ECG monitoring can be invaluable when used appropriately; a 24‐ or 48‐hour Holter is of little to no value when paroxysmal AF is expected, except when symptoms occur daily. Longer term event monitoring carries a higher diagnostic yield but is often limited by expense and patient tolerance. There are several emerging technologies with variable sensitivities and specificities, including smartphone‐based technologies. For example, this includes Kardia, implantable cardiac monitors, which can record single lead ECG tracings for a period of 3‐4 years and the Apple Watch.

The use of Apple Watch for detecting AF was supported by Perez et al,^31^ who conducted a prospective, single‐arm, open‐label, siteless study which included 419 297 individuals who had no prior history of AF and have an Apple iPhone and Apple Watch. Those individuals were followed for a median of 117 days. Out of the 419 297 individuals, 2161 received notification of irregular pulse through their smartwatches. Out of 2161, 84% of the notifications were consistent with AF, and 34% of individuals had AF on subsequent ECG patch analysis.

Additionally, there is an ongoing long‐term prospective, randomized, nationwide study conducted by Apple and Johnson & Johnson. One arm of the study will be using the Heartline app on their iPhone, whereas the other arm will be using an Apple Watch Series 5. The primary outcome of this study is to assess the relationship of using technology such as an iPhone with the Heartline app and the Apple Watch in the detection of AF while also assessing if the clinical outcome is improved, such as mitigation of stroke risk. Other desired secondary outcomes of this study consist of determining whether a heart health engagement program and medication adherence intervention would benefit those with previously diagnosed AF, understanding the overall impact of technology in identifying or managing other health problems beyond the heart, and lastly to support the advancement of clinical studies in this field of medicine where technology intersects with medicine.

## 2 FINANCIAL IMPACT OF AF

The prevalence of AF is expected to significantly increase over the next few years. This is partially explained by the improved survival of associated medical problems. Therefore, the economic burden of AF will continue to worsen in the future due to the healthcare cost for preventing and treating AF and its complications. For example, between the years 1985 and 1999, hospitalizations due to AF have doubled.^32^ Based on 2001 data, there were approximately 350 000 hospital admissions, 276 000 emergency room visits, and 5 million office visits annually in the United States for AF diagnosis and management.^32^ During that year, the estimated total cost was about $6.65 billion; 44% of which was for hospitalizations, and 4% was for prescription medications.

## 3 MANAGEMENT OF AF

Management of AF in the elderly population consists of an integrated, systematic, and multidisciplinary approach to mitigate the risk of catastrophic sequelae that may follow as a result of inadequate treatment. Patients at presentation may have new‐onset AF or may have been previously diagnosed, both of which require complete evaluation and consideration of potential risk factors that may affect the outcome of AF therapy. The management of AF should focus first and foremost on assessing the risk of thromboembolism and instituting appropriate and timely anticoagulation therapy. Beyond that, there are two approaches to the management of AF, these being a focus on either rate control or restoring and maintaining sinus rhythm (rhythm control). Close follow‐up should be maintained with a focused assessment on any change in the patient's functional, cognitive, or clinical status.

Rate versus rhythm control has been a common debate in the medical literature. A variety of studies have shown clear benefits with either therapy. Therefore, the approach instituted should be individualized, taking into account the clinical and socioeconomic factors.^33^ Furthermore, the management of AF in the elderly population requires an increased understanding of its relationship with falls and HF, two very common comorbidities that often lead to clinical confusion in the management of AF in this population.

### 3.1 Impact of AF management in the elderly population

AF is the most common arrhythmia to occur in the elderly population and has a major impact on their health. Seventy percent of patients who have AF are between the ages of 65 and 85 years old.^34^ Once diagnosed, AF should be assessed in a multidisciplinary manner, taking into consideration factors such as the patient's functional and socioeconomic status and any associated comorbidities. AF increases the risk of mortality; in the Framingham cohort, patients with AF had an odds ratio for mortality of 1.5 in male patients and 1.9 in female patients.^35^ Management should be initiated with an eye towards the prevention of thromboembolic episodes, abnormalities in cardiac function incited by arrhythmia, deterring side effects of the medications used, and improvement in the quality of life. The primary goals of AF management consist of anticoagulation to prevent thromboembolic events and rhythm or rate control. Anticoagulation with warfarin in the elderly population has been associated with a reduced risk of thromboembolism compared to aspirin.^36^ Careful observation of common comorbidities and polypharmacy should be taken into consideration when treating these patients as this can affect their long‐term outcomes. However, therapeutic intervention should not be withheld for common assumptions. Increased risk of fall and concomitant HF are two common reasons associated with withholding anticoagulation therapy. However, the benefit of anticoagulation in decreasing the risk of catastrophic thromboembolic events outweighs these risks.

### 3.2 Falls and AF management

Falls are among the major causes of morbidity and mortality in the elderly population and the second leading cause of unintentional death.^37^ Other than fall‐induced injuries, falls also increase the risk of bleeding, especially when international normalized ratio (INR) levels trend above therapeutic levels. Therefore, frequent monitoring of INR level is paramount in patients on warfarin therapy. Alternatively, subtherapeutic INR levels increase the risk of thromboembolic episodes. Hesitancy to prescribe adequate pharmacological anticoagulation for those with AF and increased risk of fall persisted and has caused physicians to withhold potentially life‐saving treatment. Although anecdotally anticoagulation was believed to lead to increased risk of bleeding and devastating outcomes in the elderly population, this has not been supported by a variety of studies. With advances in medications and further understanding of their risks and benefits within clinical trials, many pharmacological modalities used in AF treatment highly benefit the elderly population. In one study, patients with nonvalvular AF were assessed at follow‐up for their risk of intracranial hemorrhage following a history of falls. Results were consistent with no difference between those with and without anticoagulation.^38^ Another study concluded that a patient would have to fall approximately 295 times in 1 year for the bleeding event outcome to outweigh the benefit of anticoagulation.^39^ Therefore, the perception of a higher risk of falls in the elderly should not be the justification for withholding anticoagulation treatment. If pharmacological anticoagulation is absolutely contraindicated in this subgroup of patients, intervention such as left atrial appendage (LAA) closure is an alternative therapy that could be considered.^40^

### 3.3 HF and AF management

HF is a leading cause of hospital admissions, with medical costs expected to be around $30.7 billion by the year 2030.^41^ The prevalence of HF has significantly increased in the past few years due to improved survival of patients treated with guideline‐directed medical therapy. The incidence and prevalence of HF are estimated to increase by approximately 46% by the year 2030.^41^ AF is often associated with HF and is associated with increased morbidity and mortality.^11^, ^42^ Furthermore, AF can trigger acute HF exacerbations. Management of patients with AF and HF exacerbation should focus on the management of the decompensation. Functionality and staging of a patient's HF is a major contributor to the prevalence of AF in these patients. As the classification of HF progresses from stage 1 to stage 4 on the New York Heart Association (NYHA) classes, there is about a 46% increase in the prevalence of AF, which in turn can worsen the HF status.^43^

AF can incite decompensatory episodes of a patient's HF with consequent hemodynamic instability. The abnormal rhythm seen in AF may lead to a decrease in cardiac output with resulting acute decompensation, particularly in patients with HF with reduced ejection fraction. Persistent and prolonged tachycardia can also lead to tachycardia‐induced cardiomyopathy or worsen pre‐existing cardiomyopathy.^44^ The loss of atrial systole, also known as “atrial kick,” can exacerbate the inadequate filling seen in HF with preserved ejection fraction. Volume overload in patients with acute decompensated HF can lead to increased atrial stretch and subsequently trigger episodes of AF with a rapid ventricular response, which in turn can worsen diastolic filling and triggers a vicious circle of HF decompensation.

Prompt recognition of acute decompensation of HF in the setting of AF with rapid ventricular response is imperative. Timely therapy should be instituted with diuretics, vasodilators, with a focus on rate control. Patients with HF with preserved ejection fraction (HFpEF) have stiff ventricles with reduced compliance. The shortened diastolic filling time that occurs with rapid rates can result in progressive decompensation in these patients. Therefore, rate control of AF is paramount in addition to standard therapy of acute HF decompensation.

During acute decompensation, beta blockers and calcium channel blockers should be avoided until euvolemia is achieved due to the negative inotropic effects of these medications. Digoxin is effective at rate control and is particularly useful in the setting of acute decompensated HF. Careful monitoring of digoxin levels and renal function should be employed in order to avoid digoxin toxicity, given its narrow therapeutic index.

### 3.4 Cognitive impairment and AF management

Cognitive impairment is a common ailment affecting the elderly; 20% of individuals over the age of seventy have mild cognitive impairment,^45^ and over 500 000 people develop dementia on a yearly basis in the United States.^46^ Globally, there are about 7.7 million new cases every year. AF and cognitive impairment often coexist. Anticoagulation therapy in patients who have AF and dementia has shown clear benefit. In the Swedish Dementia Registry, 8096 patients were found to have concomitant AF.^47^ Of these, one third were treated with warfarin, one third with antiplatelet medications, and the remainder were treated with no anticoagulation. Patients treated with warfarin had a lower rate of ischemic stroke and death compared to patients who received antiplatelet therapy or no anticoagulation. These findings highlight the importance of anticoagulation in patients with AF regardless of the degree of cognitive impairment.

### 3.5 Frailty and its impact on treatment

Frailty is a common clinical syndrome in older adults that carries an increased risk for poor health outcomes including falls, incident disability, hospitalization, and mortality. Frailty is defined as a clinically recognizable state of increased vulnerability resulting from an aging‐associated decline in reserve and function across multiple physiologic systems. Frailty has been operationally defined by Fried et al. as meeting three out of five phenotypic criteria indicating compromised energetics: low grip strength, low energy, slowed walking speed, low physical activity, and/or unintentional weight loss.^48^

Four to sixteen percent of people above the age of 65 years suffer from frailty.^49^, ^50^ Frailty should be taken into consideration when managing chronic illnesses, as this would result in improved clinical outcomes. A holistic approach should be implemented when managing AF in the frail patient; this includes making clinical decisions regarding the optimum mode of AF control and stroke prevention.

### 3.6 Rate versus rhythm control

The optimal approach to managing AF, whether it be a focus on rate control or restoring sinus rhythm, has been long the subject of debate. A variety of studies have shown clear benefits with either therapy. Therefore, therapy should be individualized based on the clinical scenario and associated comorbidities.^33^

Rate control therapy is favored in patients over the age of 80 years old, which accounts for 35% of patients with AF.^51^ This age group is at a greater risk of adverse effects from rhythm control medications, which tend to have proarrhythmic side effects. These patients have a higher rate of long standing persistent AF and permanent AF with significant left atrial enlargement, which leads to failure of antiarrhythmic therapy in restoring sinus rhythm. Rate control is a safe and effective approach in these patients as has been shown in the AFFIRM and RACE trials, which showed no difference between a rhythm control strategy and a rate control strategy in terms of improvement in the quality of life.^52^ In fact, a rhythm control strategy has been shown to be associated with a higher rate of hospitalization.

More recently, the EAST‐AFNET 4 trial has shown that an early rhythm control strategy within the first year of AF diagnosis in patients with AF and associated cardiovascular disease is associated with improved outcomes and reduction in the risk of stroke and cardiovascular death. This is a practice changing trial that could lead to a paradigm shift in the management of AF, particularly in younger patients with cardiovascular comorbidities.^53^

Additionally, a rhythm control strategy is associated with greater exercise capacity and improved quality of life in younger patients. Patients who fail rate control therapy, HF patients, or patients with new‐onset AF can all be considered for a rhythm control strategy.^52^

### 3.7 Pharmacological anticoagulation

Thrombus development within the left atrium can be associated with disastrous outcomes, with a higher risk in those who are inadequately anticoagulated. Whether the AF is classified under subclinical, paroxysmal, persistent, or permanent, the risk of thromboembolic event persists. Even with sufficient educational counseling regarding risks of improper management of AF, low rates of pharmacological compliance have persisted especially in the elderly who have a variety of chronic disorders and other medications taken on a daily basis.^54^

The CHADSVASC 2 scoring system has been validated as a tool to assess the risk of thromboembolism in patients with AF. A score of 2 or greater indicates the necessity of initiating anticoagulation therapy, whereas a score of 1 requires clinical judgment to determine whether or not treatment should be started. Studies have shown that the annual risk of ischemic stroke with scores of 0, 1, and 2 were 0.2%, 0.6%, and 2.2% respectively.^55^ Based on the CHADSVASC 2 system, adults aged 75 years old and older are automatically assigned a score of 2; hence, anticoagulation is indicated in this group of patients. Absolute contraindications to the use of anticoagulant pharmacotherapy are quite rare, a few being major intracranial pathology or decompensated liver disease.^56^ The risk of ischemic stroke is reduced once the patient is started on anticoagulation and within the therapeutic window. The benefits of anticoagulation typically outweigh the risk of a bleeding episode^57^, ^58^ as described earlier in this article

The use of Vitamin K antagonists such as warfarin can introduce a variety of challenges when managing elderly patients. Other medications may interact with the metabolism of warfarin by interfering with the activity of the enzymes responsible for warfarin metabolism. Some of these medications are well‐known to clinicians, while some other medications have an unknown interaction profile with warfarin, presenting potential for further complications. Some of these medications can be enzyme inducers (i.e., decrease the effect of warfarin) and some can be enzyme inhibitors (ie, increase the effect of warfarin), as seen in Table 3.^59^ The clinical significance of these interactions is vital for the patient's well‐being since warfarin's therapeutic range is quite narrow. For example, a highly elevated INR may hold serious risks of bleeding events.

**TABLE 3:** List of medications that interfere with warfarin metabolism

Inducer medications	Inhibitor medications
Rifampin	Azole antifungals
Carbamazepine	Doxycycline
Phenytoin	Metronidazole
Primidone	Amiodarone
Phenobarbital	Sulfamethoxazole
Ritonavir	Testosterone
Nafcillin	Fluoroquinolones
Azathioprine	Macrolides
Sucralfate	Rosuvastatin

Although genetic variation (hepatic cytochrome P‐450 variance or Vitamin K epoxide reductase complex) in the population plays a role in varying responses to treatment with warfarin, pharmacogenetic testing is not routinely recommended. Two large meta‐analyses of randomized trials demonstrated that testing for genetic variation and incorporating this data into the dosing regimen has not reduced the amount of bleeding or thromboembolic episodes.^60^

Diet pattern may lead to abnormalities in adequate INR control. Certain foods^61^ that contain high, medium, and low amounts of Vitamin K are portrayed in Table 4. The goal seen in patients on warfarin therapy is to maintain a constant level of dietary Vitamin K intake. Avoiding a significant decrease or increase in Vitamin K containing foods may alter the INR pattern seen on the agreed warfarin dose. Therefore, this is another reason of many why INR monitoring is mandatory while on warfarin. This is also another limiting factor for warfarin use, especially in the elderly population.

**TABLE 4:** Demonstrates how many MCG of Vitamin K available in different kinds of vegetable that may interfere with Warfarin metabolism

Name of food/serving size	Amount of vitamin K
Fresh brussels sprouts, 1/2 cup	High, 110 mcg
Frozen or fresh Turnip, 1/2 cup	High, 265‐425 mcg
Fresh or frozen kale, 1/2 cup	High, 530‐565 mcg
Fresh or frozen collard, 1/2 cup	High, 530‐565 mcg
Fresh or frozen cooked spinach, 1/2 cup	High, 444‐514 mcg
Fresh or frozen cooked asparagus, 4 spears	Medium, 30‐48 mcg
Frozen cooked broccoli, 1/2 cups	Medium, 80 mcg
Raw, green or red cabbage, 1/2 cups	Medium, 14‐26 mcg
Sweet, pickle relish, 1 tablespoon	Medium, 13 mcg
Fresh or frozen cooked carrots, 1/2 cup	Medium, 10 mcg
Raw celery, 1/2 cup	Medium, 17 mcg
Fast‐food type coleslaw, 1/2 cup	Medium, 37 mcg
Romaine lettuce, 1 cup	Medium, 57 mcg
Canola oil, 1 tablespoon	Medium, 17 mcg
Frozen okra, 1/2 cup	Medium, 44 mcg
Avocado, 1 ounce	Low, <10 mcg
Chickpeas, 1/2 cup	Low, <10 mcg
Mayonnaise, 1 tablespoon	Low, <10 mcg
Olive oil, 1 tablespoon	Low, <10 mcg
Green or red peppers, 1/2 pepper	Low, <10 mcg
Potatoes, 1 potato	Low, <10 mcg
Tomatoes, 1 tomato	Low, <10 mcg

In comparison, Direct Oral Anticoagulants (DOACs) have shown superiority and better safety profile compared to warfarin, particularly in the elderly population.^62^ DOACs have been associated with lower risks of major bleeding and thromboembolic episodes.^62^ There are multiple studies that support the use of DOACs in the setting of nonvalvular AF; of them, the ARISTOTLE trial, a randomized double‐blind trial which included 18 201 patients, apixaban was compared to warfarin by looking at the incidence of stroke or systemic emboli, designated as primary outcome, and secondary objectives that observed rates of major bleeding and death from any cause.^63^ Primary outcomes occurred in 1.27% in the apixaban group versus 1.60% in the warfarin group after a median duration of 1.8‐year follow‐up. Major bleeding risk was 2.13% in the apixaban group versus 3.09% in the warfarin group, and the rates of death from any cause were 3.52% in apixaban group and 3.95% in the warfarin group. Apixaban was considered to be superior to warfarin by preventing systemic emboli and stroke, while also having a lower bleeding profile and a lower rate of death by any cause.

Additionally, a retrospective cohort study using data from November 2010 to February 2015 analyzed the risk of major bleeding in 44 057 patients while on anticoagulation therapy with warfarin versus various DOACs such as apixaban, rivoraxaban, and dabigatran.^64^ An unadjusted rate of major bleeding episodes was 2.8 per 100 person‐years with dabigatran, 3.3 with apixaban, 5.0 with rivaroxaban, and 6.0 with warfarin. This supported the conclusion that highest risk of bleeding is typically seen while on warfarin therapy. Furthermore, it did show that apixaban had a lower risk of major gastrointestinal bleeding events when compared with dabigatran. This study was consistent with many other pivotal clinical trials that have compared safety profiles of anticoagulant therapies.

Moreover, a prospective open cohort study, published in 2018, compared the risk of major bleeding, embolic events and all cause mortalities between DOACs and warfarin in patients with or without AF.^65^ In the subset of patients with AF, apixaban was associated with a lower rate of major bleeding events (adjusted hazard ratio: 0.66) and intracranial bleeding (adjusted hazard ratio: 0.40) when compared to warfarin. On the other hand, in patients without AF, apixaban was associated with a decreased risk of major bleeding (adjusted hazard ratio: 0.60) and any gastrointestinal bleeding (adjusted hazard ratio: 0.55) when compared to warfarin. Interestingly, in patients both with and without AF, rivaroxaban and lower doses of apixaban were associated with a higher risk of all‐cause mortality.

DOACs have less dietary and pharmacological interactions and do not necessitate periodic INR monitoring. DOACs are typically the preferred class of medication in the elderly population unless contraindicated. DOACs should not be used in patients with mechanical heart valves or severe mitral stenosis, noncompliance with daily medications, patients with enzyme‐modifying antiepileptic or HIV drugs, or chronic kidney disease (except Apixaban).^54^ The use of aspirin is not recommended for the prevention of thromboembolic episodes in patients with AF.^66^

### 3.8 Appendage occlusion for stroke prevention

The left atrial appendage is the site of thrombus formation in patients with AF.^67^ In certain patients who are not candidates for pharmacological anticoagulation (recurrent bleeding episodes), appendage exclusion using occluder or ligation devices could be considered,^68^ as seen in Table 5.

**TABLE 5:** Demonstrates the available left atrial occluding devices

Name of device	Type of device	Characteristics	FDA approval
WATCHMAN device	A nitinol cage that is self‐expandable implanted within the LAA. Covered by permeable polyethylene terephthalate membrane. Placed via a transseptal approach.	Placed in LAA for patients with nonvalvular AF who also have sensible reasons to not take long‐term anticoagulation therapy	Approved by the FDA in the United States in 2015
Amplatzer cardiac plug	Endovascular device constructed of nitinol mesh, including proximal left atrial disk and distal left atrial appendage lobe which have a polyester mesh	Shorter than the WATCHMAN device and may be of greater benefit in patients with a short appendage	Has not yet received FDA approval in the United States
WaveCrest device	Endovascular device containing single‐lobe nitinol based design to occlude the LAA. Covered by foam layer on the LAA side.	Can be implanted proximally in the LAA. Greater benefit when compared to WATCHMAN if the LAA is too small to accommodate deeper devices	Has not yet received FDA approval in the United States
LARIAT system	Nonsurgical, percutaneous device	Requires access to the epicardial and endocardial space. A magnetic guide would be placed within the LAA to allow the epicardial lasso to tie off the LAA. A highly valued benefit of this procedure is that no foreign body is left behind, obviating the need for anticoagulant or antiplatelet therapy post‐procedure. This device is also preferable for patients who cannot tolerate endovascular procedures. This device should not be used in patients with history of cardiac surgery or unusual left atrial appendage anatomy.	FDA approved for soft tissue closure but not for LAA occlusion. Has been reported by the FDA that complications including laceration or perforation of the heart, or complete LAA detachment from the heart have been reported with the use of the LARIAT system.

In the United States, the WATCHMAN device, a nitinol cage covered in polyethylene terephthalate, is the most commonly used device for occlusion of the left atrial appendage. This has been demonstrated in the PROTECT AF which demonstrated that the WATCHMAN device is non‐inferior to pharmacological anticoagulation with warfarin for prevention of stroke, embolization, and cardiovascular death.^68^

## 4 COMPLICATIONS OF AF

AF management is vital for maintaining the well‐being of patients. Inadequate treatment of AF, which is more frequently encountered in the elderly population, may lead to catastrophic sequelae. Complications of AF include ischemic stroke, silent cerebral ischemia, transient ischemic attack, and systemic embolization.^69^

The annual incidence of strokes in the United States is estimated to be around 795 000/year, most commonly in the elderly population.^70^, ^71^ Individualized risk factors that add to this risk profile are prior stroke or transient ischemic attack, HF, hypertension, diabetes mellitus, or older age (>65 years old). Strokes as a result of AF have been known to be associated with worse outcomes when compared to strokes from other etiologies.^72^ This is believed to be due to a greater‐sized thrombus that develops in the atrial cavity, which is relatively bigger than emboli from other sources. This finding has been supported by comparing the rates of hemispheric versus retinal events in patients with and without AF, concluding at 25:1 and 2:1, respectively.^72^

Silent cerebral ischemia may also occur which would be evident only by imaging since there is a lack of clinical manifestations. Occurrence of this finding in patients with AF reported by a meta‐analysis of 17 different studies has been shown to be about 40% when using magnetic resonance imaging (MRI).^73^ This form of cerebral ischemia is also more prevalent in patients with known diagnosis of persistent AF, which is more common in the elderly population.^74^

Patients that are currently in sinus rhythm have better overall long‐term outcomes. In the Framingham Heart Study, an episode of AF was associated with an increased risk of death even with adjustment for pre‐existing cardiovascular disease.^75^

## 5 SUMMARY

In the past few years, there have been many advances in the management of AF. However, reluctance to treat AF in the elderly population is still a prevailing issue. AF is a significant public health crisis that is more common among the elderly and the management in this population has been limited due to the hesitancy of healthcare providers to initiate these patients on adequate anticoagulation. Less than two third of the elderly population with AF are on anticoagulation, increasing their vulnerability to tragic outcomes.

There is a clear overestimation of the bleeding risk in these patients with an underestimation of the thromboembolic risk, leading to poor management. Physicians should take an individualized approach when managing the elderly patient with AF, taking into consideration the high risk of embolic stroke and the multitude of options available for stroke prevention whether through pharmacological anticoagulation or LAA occluding devices.

## CONFLICT OF INTEREST

Authors declare no conflict of interests for this article.
